# Associations of systemic inflammation and systemic immune inflammation with serum uric acid concentration and hyperuricemia risk: the mediating effect of body mass index

**DOI:** 10.3389/fendo.2024.1469637

**Published:** 2024-12-09

**Authors:** Yueyue Zhang, Shichao Han, Zhizhou Duan, Xu Tian, Xueyi Li, Guangdong Hou, Xuelin Gao, Chunjuan Tian, Xiangyu Li, Wenyuan Yu, Qin Zhou, Zhiping Niu, Fuli Wang

**Affiliations:** ^1^ Department of Urology, Xijing Hospital, Air Force Medical University, Xi’an, China; ^2^ Preventive Health Service, Jiangxi Provincial People’s Hospital, The First Affiliated Hospital of Nanchang Medical College, Nanchang, Jiangxi, China; ^3^ Department of Radiology, Baoshi Flower Changqing Hospital, Xi’an, China; ^4^ Shanghai Tenth People’s Hospital, Tongji University, Shanghai, China; ^5^ School & Hospital of Stomatology, Wuhan University, Wuhan, China; ^6^ Key Laboratory of Shaanxi Province for Craniofacial Precision Medicine Research, College of Stomatology, Xi’an Jiaotong University, Xi’an, Shaanxi, China; ^7^ Department of Environmental Health, School of Public Health, Fudan University, Shanghai, China

**Keywords:** hyperuricemia, uric acid, systemic inflammatory, systemic immuneinflammatory, body mass index, mediation effect analysis

## Abstract

**Background:**

With the development of lifestyle, elevated uric acid and hyperuricemia have become important factors affecting human health, but the biological mechanism and risk factors are still unclear.

**Methods:**

A multi-stage, cross-sectional study of 41,136 adults from the NHANES 2003-2018 was conducted. Serum uric acid concentrations, platelet, neutrophil, lymphocyte, and monocyte counts were measured. The systemic inflammation response (SIRI) index and systemic immune-inflammatory (SII) index were calculated to reflect systemic inflammation and systemic immune inflammation. The height and weight data were obtained to assess body mass index (BMI). Generalized linear models were used to examine the relationships of SIRI and SII with uric acid and hyperuricemia risk, as well as the associations of SIRI and SII with BMI, and BMI with uric acid and hyperuricemia risk. Causal mediation effect model was used to assess the mediating effect of BMI in the relationships of SIRI, and SII with uric acid concentration and hyperuricemia risk.

**Results:**

The prevalence of hyperuricemia in US adults is 19.78%. Positive associations were found in the relationships of SIRI and SII with uric acid level, hyperuricemia risk, and BMI, as well as the relationships of BMI with uric acid and hyperuricemia risk. Causal mediation effect model showed that BMI played an important mediating role in the relationships of SIRI, and SII with uric acid concentration and hyperuricemia risk, with the proportion of mediating effect ranging from 23.0% to 35.9%.

**Conclusion:**

Exposure to higher SIRI and SII is associated with increased uric acid concentration and hyperuricemia risk in adults, and BMI plays an important mediating effect. Reducing systemic inflammation and systemic immune inflammation and proper weight control could be effective ways to reduce hyperuricemia prevalence and related health problems.

## 1 Introduction

Uric acid is a metabolite derived from the metabolic breakdown of purines in the human body, predominantly eliminated through renal excretion. Elevated levels of uric acid can result in the occurrence of hyperuricemia (1, 2). As a metabolic disorder, the increasing prevalence of hyperuricemia has emerged as a significant global public health concern (3–5). During the process of rapid urbanization, the alteration of residents’ modern lifestyles, changes in dietary patterns, and the escalation of obesity rates are considered important risk factors contributing to the elevation of uric acid concentration and the increased prevalence of hyperuricemia (1, 6). Previous studies indicated that the prevalence of hyperuricemia in US adults was 20.1%, affecting approximately 38 million individuals (7). Abnormally elevated uric acid concentration and hyperuricemia have been confirmed as the pathological basis of gout. Gout is an inflammatory joint disease caused by the deposition of uric acid crystals in the joints, often characterized by joint swelling, pain, and impaired mobility in affected individuals (8). Moreover, hyperuricemia is closely associated with the occurrence and progression of other diseases, such as hypertension, diabetes, chronic kidney disease, and cardiovascular diseases (4, 9, 10).

Although the specific biological mechanisms underlying the elevation of uric acid concentration and hyperuricemia prevalence have not been fully elucidated, systemic inflammation and immune dysregulation are believed to be important regulatory mechanisms of uric acid concentration (11–14). Recent studies indicated that exposure to higher systemic inflammation response index (SIRI) and systemic immune-inflammation index (SII) was associated with increased uric acid concentration and hyperuricemia risk (12, 15). For example, a cross-sectional study of 5,568 US adolescents found that exposure to higher SII levels was related to increased serum uric acid concentration and hyperuricemia risk (12). A cross-sectional study of 8,095 Chinese adults reported a linear positive association between SIRI and prevalent hyperuricemia (15). However, to our knowledge, the mechanisms underlying the positive relationships of SIRI, and SII with uric acid concentration and hyperuricemia risk remain unclear. Body mass index (BMI), as a common indicator of obesity and metabolic dysfunction (16, 17), has been reported to be significantly associated with inflammatory response (18) and elevated uric acid concentration (19–21). Moreover, previous studies indicated that exposure to higher systemic inflammation and immune inflammation may increase body weight through various biological mechanisms, leading to elevated uric acid concentration (22–24). These mechanisms include the release of inflammatory cytokines causing insulin resistance (23, 25, 26) and adipose tissue inflammation (25), exacerbation of oxidative stress (27), and dysbiosis of gut microbiota (28, 29). Based on the above findings, we hypothesized that BMI could play an important mediating role in the relationships of SIRI, and SII with uric acid and hyperuricemia risk. To the best of our knowledge, no study has examined the potential mediating effect of BMI in the relationships of SIRI, and SII with uric acid or hyperuricemia risk.

In this multi-stage, cross-sectional study, we investigated the relationships between SIRI, and SII with uric acid concentration and hyperuricemia risk. Then, the mediating effect of BMI in the relationship of SIRI, and SII with uric acid concentration and hyperuricemia risk was estimated. This study aims to identify key biological mechanisms underlying hyperuricemia and provide potential intervention strategies.

## 2 Methods

### 2.1 Study population

The study participants were derived from the National Health and Nutrition Examination Survey (NHANES), which is an ongoing cross-sectional study that investigates a nationally representative sample of adults and children in the US. The NHANES study conducts surveys on approximately 5,000 US participants from 15 counties annually, with each survey cycle spanning two years. In this study, we included a total of 80,132 participants from eight NHANES follow-up surveys conducted between 2003 and 2018. Inclusion criteria were as follows (1): participation in standard blood biochemistry testing with complete serum uric acid data; (2) participation in complete blood cell count with no missing data for platelet, neutrophil, lymphocyte, and monocyte counts; (3) participation in physical examination with no missing data for height and weight; (4) individuals aged 18 years and above.

The NHANES study received approval from the National Center for Health Statistics Ethics Review Board (https://www.cdc.gov/nchs/nhanes/irba98.htm), and informed consent was obtained from all participants.

### 2.2 SIRI and SII measurement

Venous blood samples were obtained from each participant and analyzed using an automated hematology analyzer (Coulter DxH 800 analyzer) for complete blood cell count (reported as 1000 cells/µL). Based on previous literature, SIRI and SII indices were calculated using platelet, neutrophil, lymphocyte, and monocyte counts (11, 30, 31). The calculation formulas for SIRI and SII are as follows:


SIRI = (neutrophil count × monocyte count) / lymphocyte count

SII = platelet count × neutrophil count/lymphocyte count

### 2.3 Serum uric acid level measurement and definition of hyperuricemia

Approximately two-thirds of the study participants underwent standard blood biochemistry testing, and serum uric acid concentration was measured using the timed endpoint method (32). Information on sample collection and processing, quality control, and quality assurance can be found in the NHANES Laboratory Procedures Manual. Details on the analytical methods, principles, and operating procedures are provided in the NHANES Laboratory Methods Document. Hyperuricemia was diagnosed when serum uric acid concentrations were ≥ 416 μmol/L (7.0 mg/dL) in males and ≥ 357 μmol/L (6.0 mg/dL) in females (1, 2, 33).

### 2.4 BMI measurement

During each follow-up visit, physical examinations were conducted on the study participants to record their weight and height measurements. The body mass index (BMI) was calculated using the following formula: BMI = Weight (kg)/(Height (m)^2).

### 2.5 Covariate

The inclusion of covariates was based on previous NHANES studies on uric acid and hyperuricemia (7, 11, 34–39), and directed acyclic graph analysis was performed to explore the potential pathways of covariates in the relationships between SIRI, SII, BMI, and uric acid concentration, and the risk of hyperuricemia (**Supplementary Figure S1**) (11). These covariates included: (1) sociodemographic factors: age (34–36), sex (7, 34), race (7, 34, 35); (2) socioeconomic factors: marital status (7), education level (7, 34); (3) dietary factors: consumption of fish and shellfish (1, 32, 38); (4) lifestyle and behavioral habits related to uric acid metabolism: smoking status (7, 35, 39), alcohol consumption (34, 35, 37), and physical activity (11, 34). Standardized questionnaires were used to collect information on the above covariates from the study participants. The intake of seafood products was assessed by surveying the consumption of shellfish and fish in the past 30 days (7). Smokers were defined as individuals who had smoked a cumulative total of 100 or more cigarettes. Alcohol consumption was defined as drinking at least once a month (2017-2018) or more than 12 times a year (2003-2016) (11). Based on the 2008 Physical Activity Guidelines for Americans, participants were categorized into four groups: “high”, “moderate”, “insufficient” and “sedentary” (40, 41).

### 2.6 Statistical analysis

Descriptive statistics were used. Mean ± standard deviation (SD) was used to represent normally distributed continuous variables, while median (P25, P75) was used to describe the non-normally distributed continuous variables. Categorical variables were presented as frequency (percentage, %). To compare the differences between non-hyperuricemia and hyperuricemia groups, Student t-tests were used for normally distributed continuous variables, while Mann-Whitney U tests were employed for non-normally distributed continuous variables. For categorical variables, chi-square tests were used to compare the differences between the non-hyperuricemia and hyperuricemia groups.

The associations of SIRI, and SII with uric acid, and hyperuricemia risk were analyzed using generalized linear models. Specifically, linear regression models were used to analyze the relationship between SIRI, SII, BMI, and uric acid concentration, while logistic regression models were utilized to assess the association between SIRI, SII, BMI, and the risk of hyperuricemia. The effect sizes were reported as the association between the SIRI, SII, and BMI with each interquartile range (IQR) increase in uric acid concentration and the risk of hyperuricemia. To adjust for potential confounding factors and test the robustness of the results, the study established a crude model and two adjusted models. Based on previous research on uric acid, sociodemographic factors (age, sex, race), socioeconomic factors (marital status, education level), dietary factors (consumption of fish and shellfish) (1, 32), and other lifestyle and behavioral habits related to uric acid metabolism (smoking status, alcohol consumption, physical activity) (34) were included as covariates in the analysis.

Causal mediation effect model was conducted to evaluate whether BMI mediates the relationships of SIRI and SII with uric acid concentration hyperuricemia risk (16, 42). Briefly, in addition to observing positive associations of the SIRI index, SII index with uric acid concentration, hyperuricemia risk (exposure-outcome), the associations of SIRI index, SII index with BMI (exposure-mediator), and the relationships of BMI with uric acid concentration, hyperuricemia risk (mediator-outcome) were examined utilizing generalized linear models. If all the associations mentioned above were statistically significant (exposure-outcome, exposure-mediator, and mediator-outcome relationships), causal mediation effect model was used to estimate the percentage of the mediating effect of BMI in the relationship of SIRI index, SII index with uric acid concentration and hyperuricemia risk (42, 43).

The statistical analysis was conducted using R software (version 4.3.2). The causal mediation effect model was conducted utilizing the “mediation” R package. A significance level of *p < 0.05* was considered as statistical significance.

## 3 Results

### 3.1 Basic characteristics of study participants

A total of 41,136 adults from NHANES 2003-2018 were included in this study. The mean age of the study participants was 47.85 ± 18.99 years. A total of 8,136 individuals were identified as having hyperuricemia, with a prevalence rate of 19.78%. The basic characteristics of the study participants are presented in **Table 1**.

**Table 1:** Basic characteristics of study subjects.

	Total (n=41,136)	Non-hyperuricemia (n=33000)	Hyperuricemia (n=8136)	*P-value*
**Age (years), mean ± SD**	47.85 ± 18.99	46.50 ± 18.71	53.35 ± 19.12	<0.001
**BMI (kg/m^2^), mean ± SD**	28.15 ± 6.43	27.40 ± 6.01	31.18 ± 7.13	<0.001
**Sex, n (%)**				<0.001
Male	20187 (49.1)	15632 (47.4)	4555 (56.0)	
Female	20949 (50.9)	17368 (52.6)	3581 (44.0)	
**Rece, n (%)**				<0.001
Mexican American	6509 (15.8)	5599 (17.0)	910 (11.2)	
Other Hispanic	3527 (8.6)	3003 (9.1)	524 (6.4)	
Non-Hispanic White	17965 (43.7)	14196 (43.0)	3769 (46.3)	
Non-Hispanic Black	8793 (21.4)	6736 (20.4)	2057 (25.3)	
Other Race - including multi-racial	4342 (10.6)	3466 (10.5)	876 (10.8)	
**Educational Level, n (%)**				<0.001
Lower than high school	14163 (34.4)	11064 (33.5)	3099 (38.1)	
High school	10141 (24.7)	8030 (24.3)	2111 (25.9)	
College graduate or above	16794 (40.8)	13878 (42.1)	2916 (35.8)	
Missing	38 (0.1)	28 (0.1)	10 (0.1)	
**Marital status, n (%)**				<0.001
Married	20244 (49.2)	16283 (49.3)	3961 (48.7)	
Widowed	3209 (7.8)	2218 (6.7)	991 (12.2)	
Divorced	4157 (10.1)	3239 (9.8)	918 (11.3)	
Separated	1223 (3.0)	993 (3.0)	230 (2.8)	
Never married	7719 (18.8)	6383 (19.3)	1336 (16.4)	
Living with partner	3059 (7.4)	2254 (6.8)	505 (6.2)	
Missing	1525 (3.7)	1330 (4.0)	195 (2.4)	
**Smoking status, n (%)**				<0.001
Yes	17701 (43.0)	1559 (4.7)	209 (2.6)	
No	21667 (52.7)	13854 (42.0)	3847 (47.3)	
Missing	1768 (4.3)	17587 (53.3)	4080 (50.1)	
**Drinking status, n (%)**				<0.001
Yes	26731 (65.0)	3933 (11.9)	740 (9.1)	
No	9732 (23.7)	21321 (64.6)	5410 (66.5)	
Missing	4673 (11.4)	7746 (23.5)	1986 (24.4)	
**Fish consumption, n (%)**				<0.001
Yes	24984 (60.7)	19947 (60.4)	5037 (61.9)	
No	10395 (25.3)	8555 (25.9)	1840 (22.6)	
Missing	5557 (13.5)	4495 (13.6)	1259 (15.5)	
**Shellfish consumption, n (%)**				<0.001
Yes	18589 (45.2)	14964 (45.3)	3625 (44.6)	
No	16799 (40.8)	13541 (41.0)	3258 (40.0)	
Missing	5531 (13.4)	4459 (13.5)	1253 (15.4)	

Statistical analyses were conducted to compare differences between non-hyperuricemia and hyperuricemia adults using Student t-tests for normally distributed continuous variables and Mann-Whitney U tests for non-normally distributed continuous variables. Chi-square tests were utilized for categorical variables to compare differences between non-hyperuricemia and hyperuricemia adults.

### 3.2 Comparison of SIRI, SII, and BMI between adults with hyperuricemia and without hyperuricemia

**Figure 1:**
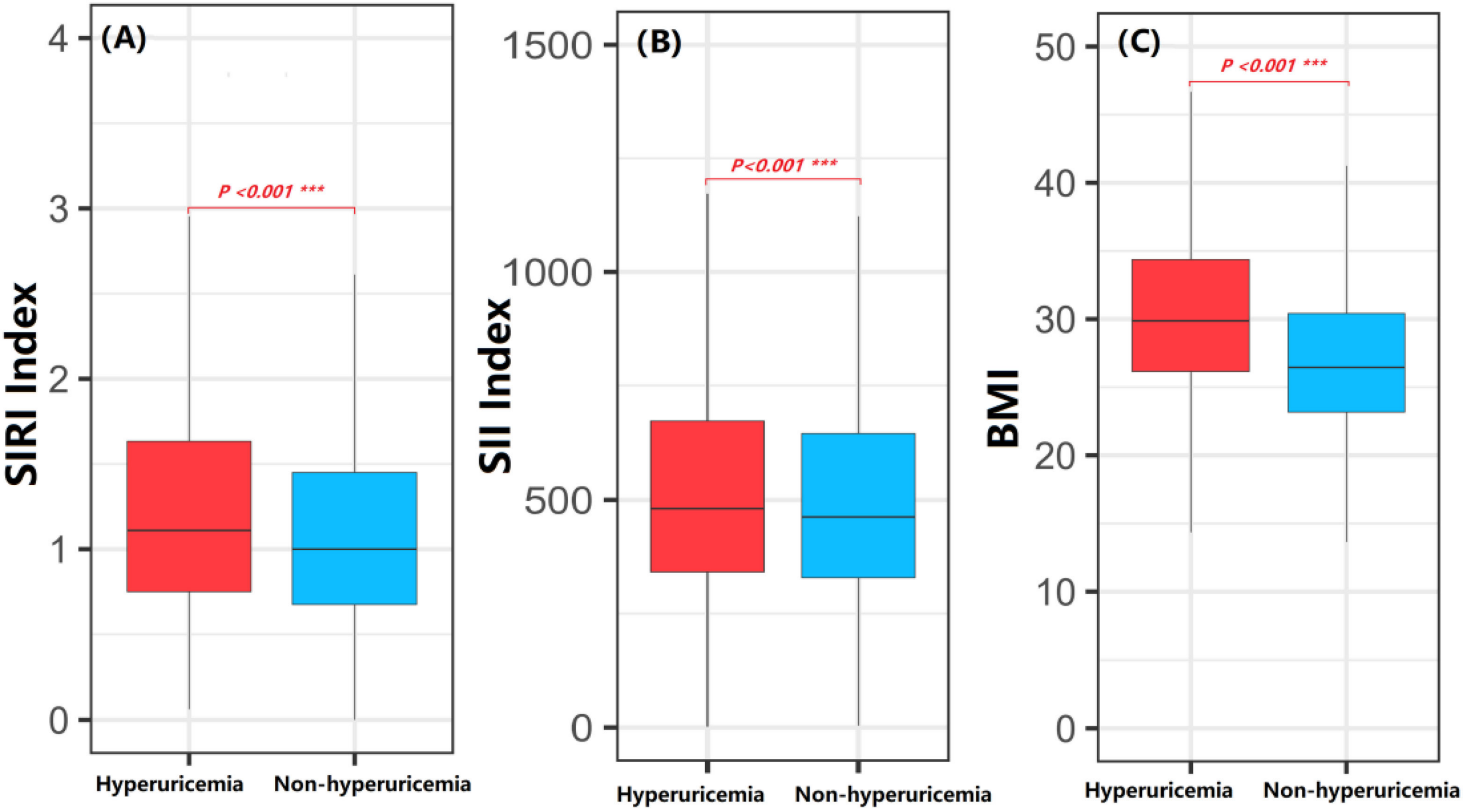
Comparison of SIRI index, SII index and BMI between hyperuricemia and non-hyperuricemia participants. Student t-tests and Mann-Whitney U tests were employed to compare the differences in SIRI index, SII index and BMI between the non-hyperuricemia and hyperuricemia groups. **(A)** SIRI index; **(B)** SII index; **(C)** BMI; The P value represents the statistical significance for testing the difference between hyperuricemia and non-hyperuricemia participants. ***P-value < 0.001.

The distribution of the SIRI index, SII index, and BMI between adults with hyperuricemia and those without hyperuricemia was compared using box plots. The SIRI index, SII index, and BMI in the hyperuricemia group were significantly higher than those in the non-hyperuricemia group (*p < 0.001*) (**Figure 1**).

### 3.3 Associations of SIRI and SII with Uric acid concentration and hyperuricemia risk

**Figure 2:**
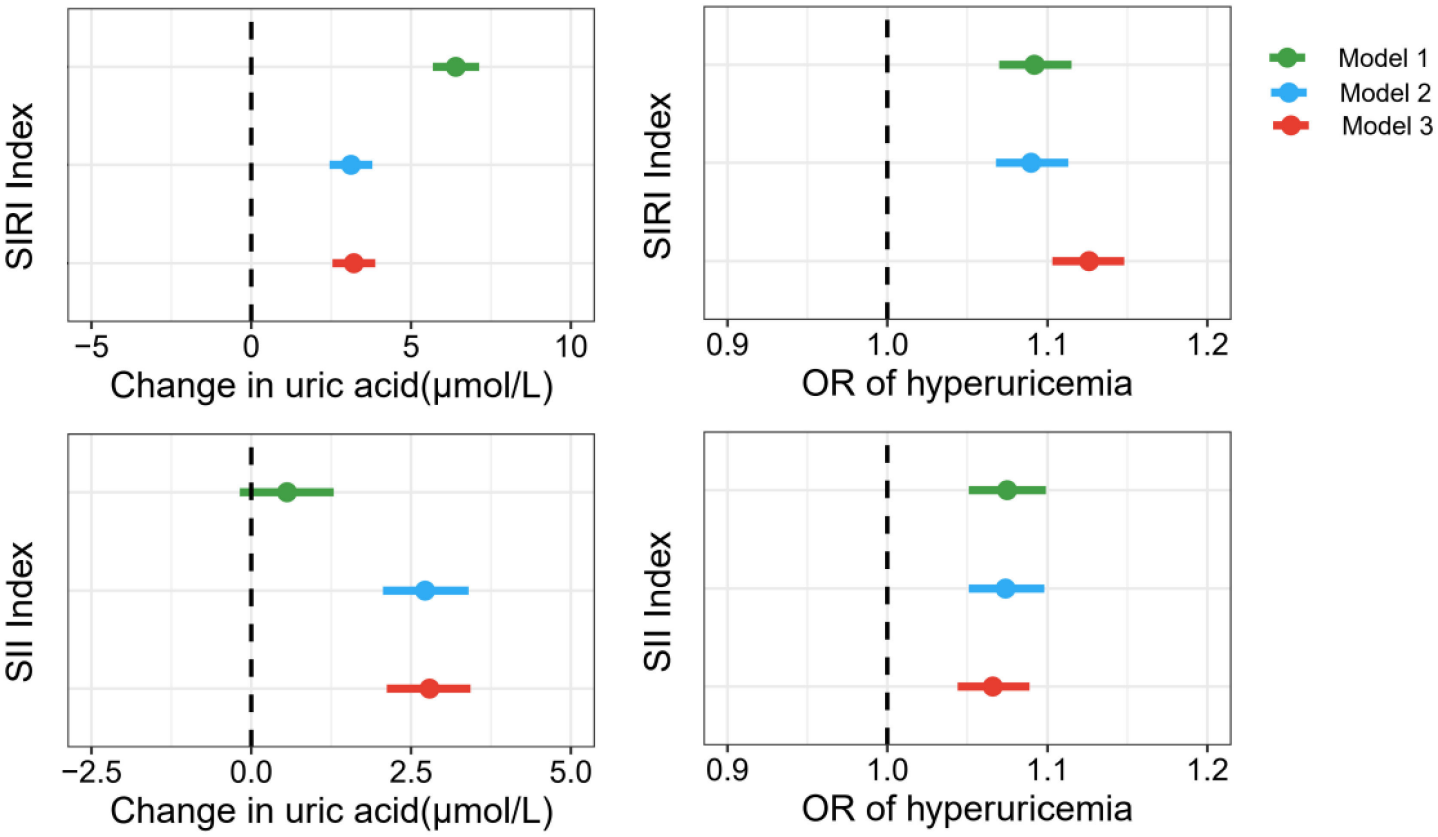
Associations of SIRI, SII with uric acid concentration and hyperuricemia risk. Model 1, unadjusted for covariates; Model 2, adjusted for age, sex, race, marital status, and education level; Model 3, adjusted for age, sex, race, marital status, education level, smoking status, alcohol consumption, and intake of fish and shellfish.

The associations of SIRI and SII with uric acid and hyperuricemia risk are presented in **Figure 2**; **Supplementary Table S1**. Except for the non-significant association between the SII index and uric acid concentration in the crude model, positive associations were all observed for the relationships of SIRI and SII with uric acid concentration. After adjusting for age, sex, race, marital status, education level, smoking status, alcohol consumption, and the intake of fish and shellfish, each IQR increase in SIRI (IQR = 0.83) and SII (IQR = 333.15) was associated with an increase of 3.21μmol/L (95%CI: 2.54, 3.88) and 2.79 μmol/L (95%CI: 2.12, 3.43) in uric acid concentration, respectively. Regarding the risk of hyperuricemia, both the crude and adjusted models indicated that the SIRI and SII were positively associated with an increased risk of hyperuricemia. After adjusting for covariates, the study found that each IQR increase in the SIRI and SII was associated with a 9.2% (OR=1.092; 95%CI: 1.070, 1.115) and 7.5% (OR=1.075; 95%CI: 1.051, 1.099) increase in hyperuricemia risk, respectively.

### 3.4 Associations of SIRI, SII with BMI

**Figure 3:**
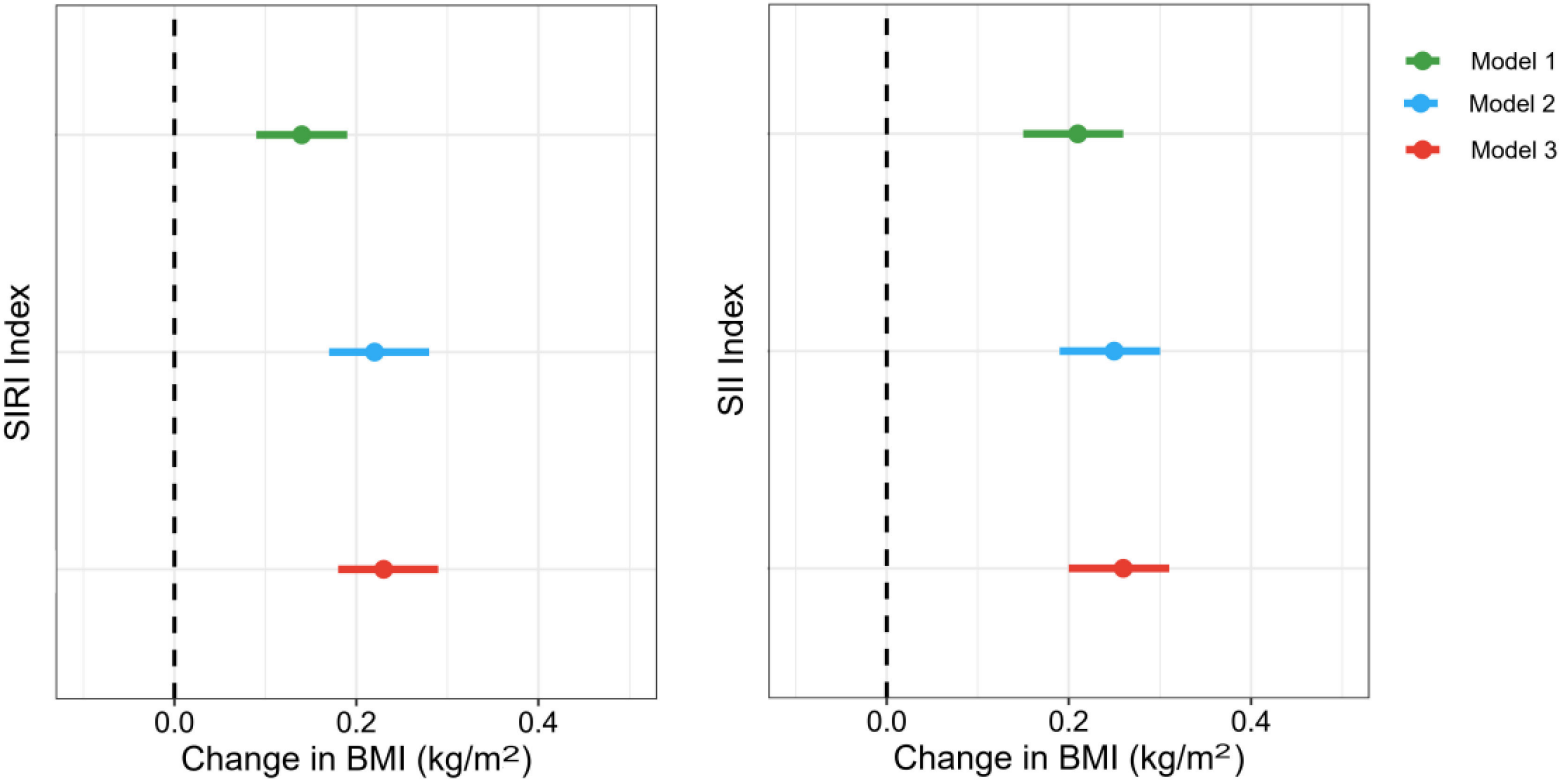
Associations of SIRI, SII with BMI. Model 1, unadjusted for covariates; Model 2, adjusted for age, sex, race, marital status, and education level; Model 3, adjusted for age, sex, race, marital status, education level, smoking status, alcohol consumption, and intake of fish and shellfish.

The associations of SIRI and SII with BMI are presented in **Figure 3**; **Supplementary Table S2**. Both crude and adjusted models showed positive relationships of SIRI and SII with BMI. After adjusting for the potential covariates, each IQR increase in SIRI and SII was associated with an increase of 0.23 kg/m^2^ (95%CI: 0.18, 0.29) and 0.26 kg/m^2^ (95%CI: 0.20, 0.31) in BMI, respectively.

### 3.5 Associations of BMI with uric acid concentration and hyperuricemia risk

**Figure 4:**
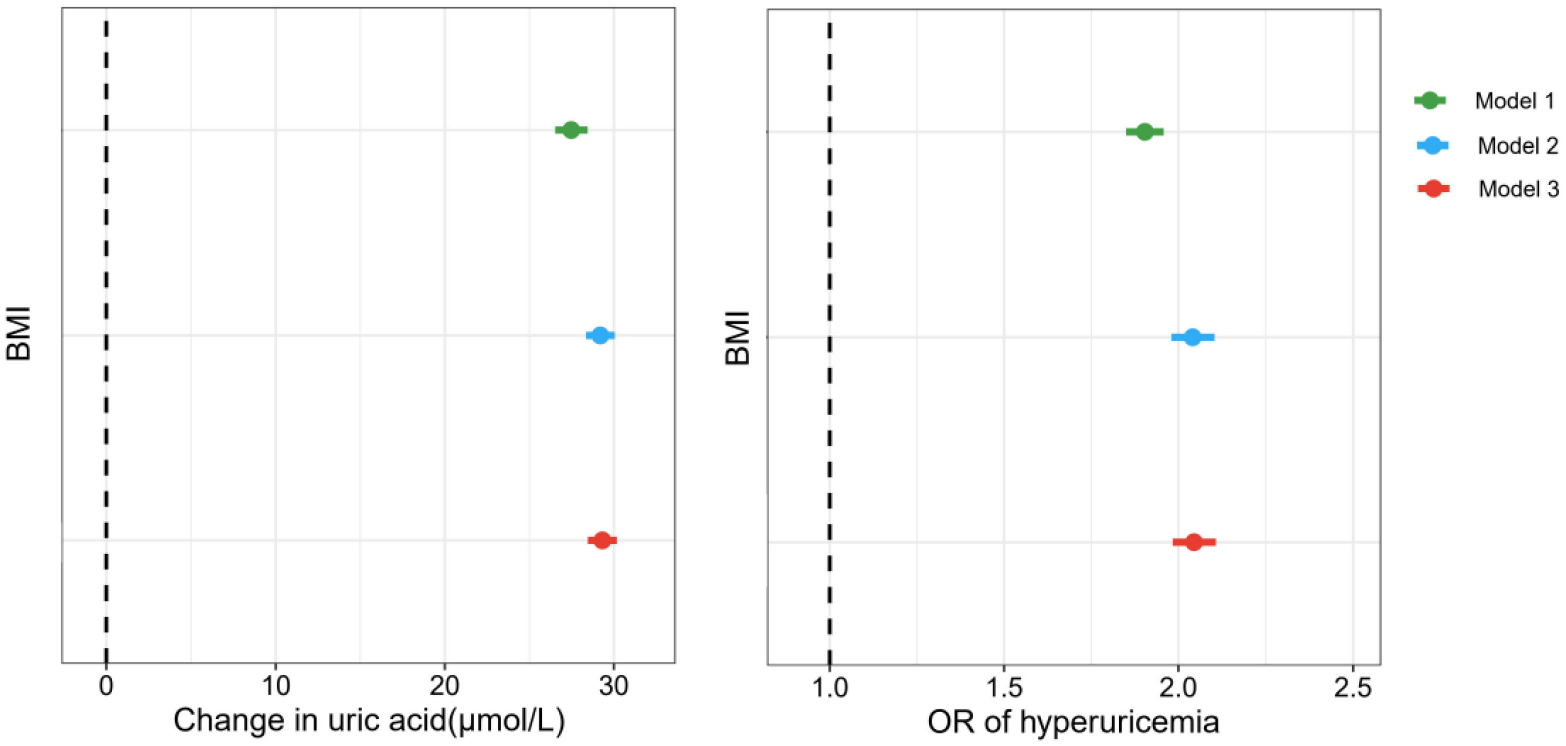
Associations of BMI with uric acid concentration and hyperuricemia risk. Model 1, unadjusted for covariates; Model 2, adjusted for age, sex, race, marital status, and education level; Model 3, adjusted for age, sex, race, marital status, education level, smoking status, alcohol consumption, and intake of fish and shellfish.

The associations of BMI with uric acid concentration and hyperuricemia risk are shown in **Figure 4**; **Supplementary Table S3**. The results indicated positive relationships between BMI and uric acid concentration, as well as hyperuricemia risk. After adjusting for covariates, each IQR increase in BMI (IQR=7.69 kg/m^2^) was associated with an increase of 29.32 μmol/L (95%CI: 28.46, 30.18) in uric acid concentration and a 104.5% (OR=2.045; 95%CI: 1.984, 2.108) increase in the risk of hyperuricemia (*p < 0.001*).

### 3.6 Mediating effect of BMI in the associations of SIRI and SII with uric acid concentration and hyperuricemia risk.

**Figure 5:**
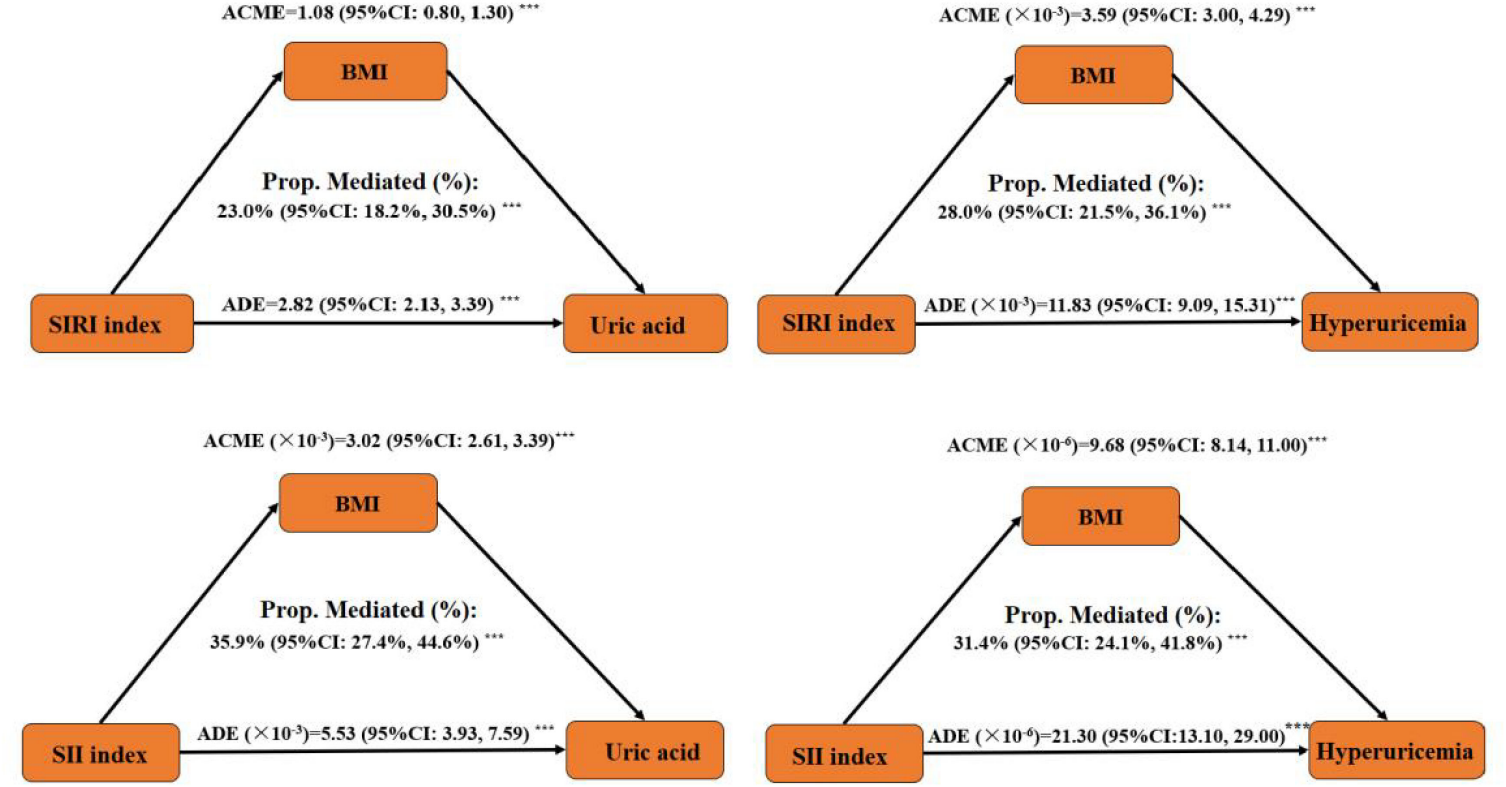
Mediation effect of BMI on the associations of SIRI, SII with uric acid concentration and hyperuricemia risk. ACME refers to the indirect effect, ADE refers to the direct effect, and the mediation percentage represents the proportion of the indirect effect to the total effect (sum of indirect and direct effects). ^***^ *P-value <*0.001.

The mediating effect of BMI in the association of SIRI, and SII, with uric acid concentration and hyperuricemia risk was analyzed, and the results are shown in **Figure 5**. The results revealed significant mediating effects of BMI in the relationships of SIRI and SII with uric acid concentration and hyperuricemia risk. The mediated proportions of BMI in the relationships of SIRI with uric acid and hyperuricemia risk were 23.0% (95%CI: 18.2%, 30.5%) and 28.0% (95%CI: 21.5%, 36.1%), respectively. The mediated proportions of BMI were 35.9% (95%CI: 27.4%, 44.6%) and 31.4% (95%CI: 24.1%, 41.8%) for the relationships of SII with uric acid and hyperuricemia risk.

## 4 Discussion

This multi-stage, cross-sectional study of 41,136 adults from the NHANES 2003-2018 conducted a comprehensive investigation on the relationship among SIRI, SII, BMI, uric acid concentration, and hyperuricemia risk. This study revealed that exposure to higher SIRI, and SII was associated with increased uric acid concentration and hyperuricemia risk, and BMI was identified as a crucial mediation factor in the relationships of SIRI, and SII with uric acid concentration and hyperuricemia risk. To our knowledge, this study might be the first study that assessed the mediating role of BMI in the relationships of SIRI, and SII with uric acid concentration and hyperuricemia risk. With the global rapid increase in uric acid concentration and hyperuricemia prevalence, our study highlights the importance of reducing SIRI and SII, maintaining body weight in lowering the prevalence of hyperuricemia, and the adverse health effects caused by high uric acid concentration.

SII and SIRI, calculated from platelets, neutrophils, lymphocytes, and monocytes, have garnered widespread attention as novel inflammatory markers in recent years. Previous studies indicated that SIRI and SII could provide a comprehensive reflection of the immune-inflammatory status of the body and have significant predictive value for various diseases, including cardiovascular diseases (12). In the current study, we found positive associations of SIRI and SII with uric acid concentration and hyperuricemia risk in a representative US population. Our findings could be supported by existing research. For example, Chen et al. conducted a cross-sectional study on 8,095 adults from the Northeast Rural Cardiovascular Health Study in 2012-2013, and found a positive association between the SIRI index and the risk of hyperuricemia, suggesting the significant value of SIRI in risk stratification and prevention of hyperuricemia (15). Xie et al. conducted a cross-sectional study on 5,568 adolescents from NHANES 2009-2018 and reported positive associations between the SII index and uric acid concentration as well as the risk of uric acid elevation (12). In addition to SIRI and SII, C-reactive protein (CRP) is also frequently used as a biomarker of systemic inflammation (44). Previous studies have reported positive associations of CRP with uric acid concentration and hyperuricemia, which partially supports our findings. For example, a cross-sectional study on obese children in adolescence found a positive correlation between CRP concentration in serum and uric acid concentration (24). To our knowledge, this study might be the largest epidemiological study that investigated the relationship of SIRI and SII with uric acid concentration and hyperuricemia risk, which could provide more comprehensive epidemiological evidence for the relationships between SIRI, SII, and uric acid and the risk of hyperuricemia.

Moreover, this study found that BMI plays an important mediating role in the relationships of SIRI, and SII with the elevation of uric acid concentration and hyperuricemia risk. Some existing findings provide supportive evidence for our results. On the one hand, BMI, as an indicator of obesity, is closely associated with uric acid concentration and hyperuricemia risk (19–21). A study of 39,736 Chinese adults from Jiangsu Province found that uric acid concentration linearly increased with BMI, and obese patients had significantly higher uric acid concentrations than underweight patients. Compared to individuals with low weight, overweight individuals had an approximately 2.98 times higher risk of hyperuricemia, and obese individuals had an approximately 5.96 times higher risk of hyperuricemia (20). Moreover, BMI levels in childhood can also affect serum uric acid concentration in adulthood, exerting long-term effects on health. A study of 298 children from Japan found that rapid BMI increase in childhood was related to a significant increase in serum uric acid concentration in adulthood (19). On the other hand, multiple studies showed that exposure to higher SIRI and SII was associated with increased BMI levels (18, 45). Wang et al. conducted a cross-sectional study of 7,420 rural residents and reported a positive association between SIRI index and obesity risk (45). Chen et al. conducted a cross-sectional study of 9,301 participants from NHANES 2005-2018 and reported positive associations of SII index with BMI and waist circumference (18). To the best of our knowledge, this study may be the first study assessing the mediating effect of BMI in the association of SIRI, and SII with the elevation of uric acid level and hyperuricemia risk, and our finding indicated that controlling weight could be an effective measure to reduce the risk of hyperuricemia and adverse health effects that caused by high uric acid concentration.

Although the exact mechanism explaining the mediating effect of BMI on the positive relationships of SIRI, and SII with uric acid concentration and hyperuricemia risk remains uncertain, some potential biological mechanisms should be paid particular attention to. Firstly, the release of inflammatory cytokines may result in insulin resistance (46), disrupting insulin signaling and affecting glucose metabolism and lipid accumulation, promoting fat deposition and obesity development (18, 47–49). Secondly, prolonged inflammation status could trigger an inflammatory response in adipose tissue, leading to dysfunction of adipocytes, affecting lipid metabolism (50) and hormone secretion (51, 52), further promoting obesity formation (53). Thirdly, oxidative stress may also play a role by disrupting cell structure and function, exacerbating tissue damage and metabolic abnormalities (54, 55). Finally, the inflammatory status may also influence the balance of gut microbiota, leading to dysbiosis, affecting energy metabolism and nutrient absorption, thereby impacting weight control and uric acid metabolism (29, 56, 57). These complex biological mechanisms interact with each other and contribute to the elevation of BMI. The increased BMI could affect uric acid metabolism through various mechanisms (58). Firstly, obesity is often accompanied by insulin resistance, which leads to reduced excretion of uric acid by the kidneys, resulting in the accumulation of uric acid in the bloodstream (59). Secondly, individuals with obesity commonly consume an excessive number of high-purine foods, thereby increasing the body’s production of uric acid (60). Finally, the increased renal workload caused by obesity impairs the kidneys’ ability to eliminate uric acid effectively (61). These factors collectively contribute to the elevation of serum uric acid concentration and hyperuricemia risk.

Our study may have some strengths. First, this study might be the largest epidemiological study that investigated the relationship of SIRI and SII with uric acid concentration and hyperuricemia risk, which could provide more comprehensive epidemiological evidence for the relationships between SIRI, SII, and uric acid and the risk of hyperuricemia. Second, this study may be the first study assessing the mediating effect of BMI in the association of SIRI, and SII with the elevation of uric acid level and hyperuricemia risk, and our finding indicated that controlling weight could be an effective measure to reduce the risk of hyperuricemia and adverse health effects that caused by high uric acid concentration. However, several limitations should be noted in our study. First, although this study included 8 waves of NHANES data from 2003 to 2018, the cross-sectional study design cannot establish causal relationships between SIRI, SII, BMI, uric acid concentration, and the risk of hyperuricemia. Secondly, despite incorporating various covariates to adjust for potential confounders in our analysis, there remains a possibility that unmeasured confounding variables could introduce some bias to our findings. Finally, our study was conducted in a representative population of US adults. Further research is warranted in diverse populations, including individuals of different ethnicities and from developing countries.

## 5 Conclusion

Exposure to higher SIRI and SII was associated with increased serum uric acid concentration and hyperuricemia risk in adults. BMI played an important mediating role in the relationships of SIRI, and SII with uric acid concentration and hyperuricemia risk. With the increasing prevalence of hyperuricemia worldwide and the rapid rise in disease burden due to elevated uric acid concentration, this study suggests that reducing SIRI and SII and maintaining body weight could be effective measures to reduce the risk of hyperuricemia and adverse health effects caused by high uric acid concentration. In the future, longitudinal studies should be conducted to validate our findings and establish causal relationships between SIRI, and SII with uric acid and hyperuricemia risk.

## Data Availability

Publicly available datasets were analyzed in this study. This data can be found here: https://www.cdc.gov/nchs/nhanes/index.htm.
