# Supplementary material for: Associations of systemic inflammation and systemic immune inflammation with serum uric acid concentration and hyperuricemia risk: the mediating effect of body mass index
Source: Front Endocrinol (Lausanne). 2024 Dec 9;15:1469637. doi: 10.3389/fendo.2024.1469637 (PMC11667560; doi:10.3389/fendo.2024.1469637)
Supplement: Supplementary file 1 [file DataSheet1.docx]

**Supplementary Material**

**Table S1. Associations of SIRI, SII with uric acid levels and hyperuricemia risk**

| **Inflammation index** | Uric acid (μmol/L) | |  | Hyperuricemia risk | |
| --- | --- | --- | --- | --- | --- |
|  | β and 95%CI | *P-value* |  | OR and 95%CI | *P-value* |
| **SIRI index (IQR=****0.83)** |  |  |  |  |  |
| Model 1 | 6.40 (5.68, 7.13) | <0.001 ^***^ |  | 1.126 (1.103, 1.148) | <0.001 ^***^ |
| Model 2 | 3.12 (2.45, 3.79) | <0.001 ^***^ |  | 1.090 (1.068, 1.113) | <0.001 ^***^ |
| Model 3 | 3.21 (2.54, 3.88) | <0.001 ^***^ |  | 1.092 (1.070, 1.115) | <0.001 ^***^ |
| **SII index (IQR=333.15)** |  |  |  |  |  |
| Model 1 | 0.56 (-0.18, 1.29) | 0.140 |  | 1.066 (1.044, 1.089) | <0.001 ^***^ |
| Model 2 | 2.72 (2.06, 3.40) | <0.001 ^***^ |  | 1.074 (1.051, 1.098) | <0.001 ^***^ |
| Model 3 | 2.79 (2.12, 3.43) | <0.001 ^***^ |  | 1.075 (1.051, 1.099) | <0.001 ^***^ |

Note: Model 1, unadjusted for covariates; Model 2, adjusted for age, sex, race, marital status, and education level; Model 3, adjusted for age, sex, race, marital status, education level, smoking, alcohol consumption, and intake of fish and shellfish.

**Table S2.** Associations of SIRI, SII with BMI

| **Inflammation index** | **BMI (kg/m^2^)** | |
| --- | --- | --- |
|  | β and 95%CI | *P-value* |
| **SIRI index （IQR=0.83）** |  |  |
| Model 1 | 0.14 (0.09, 0.19) | <0.001 ^***^ |
| Model 2 | 0.22 (0.17, 0.28) | <0.001 ^***^ |
| Model 3 | 0.23 (0.18, 0.29) | <0.001 ^***^ |
| **SII index （IQR=333.15）** |  |  |
| Model 1 | 0.21 (0.15, 0.26) | <0.001 ^***^ |
| Model 2 | 0.25 (0.19, 0.30) | <0.001 ^***^ |
| Model 3 | 0.26 (0.20, 0.31) | <0.001 ^***^ |

Note: Model 1, unadjusted for covariates; Model 2, adjusted for age, sex, race, marital status, and education level; Model 3, adjusted for age, sex, race, marital status, education level, smoking, alcohol consumption, and intake of fish and shellfish.

**Table S3.** Associations of BMI with uric acid levels and hyperuricemia risk

| **BMI (IQR=7.69 kg/m^2^)** | Uric acid (μmol/L) | |  | Hyperuricemia risk | |
| --- | --- | --- | --- | --- | --- |
|  | β and 95%CI | *P-value* |  | OR and 95%CI | *P-value* |
| Model 1 | 27.48 (26.53, 28.45) | <0.001 ^***^ |  | 1.904 (1.850, 1.958) | <0.001 ^***^ |
| Model 2 | 29.20 (28.35, 30.06) | <0.001 ^***^ |  | 2.041 (1.980, 2.104) | <0.001 ^***^ |
| Model 3 | 29.32 (28.46, 30.18) | <0.001 ^***^ |  | 2.045 (1.984, 2.108) | <0.001 ^***^ |

Note: Model 1, unadjusted for covariates; Model 2, adjusted for age, sex, race, marital status, and education level; Model 3, adjusted for age, sex, race, marital status, education level, smoking, alcohol consumption, and intake of fish and shellfish.


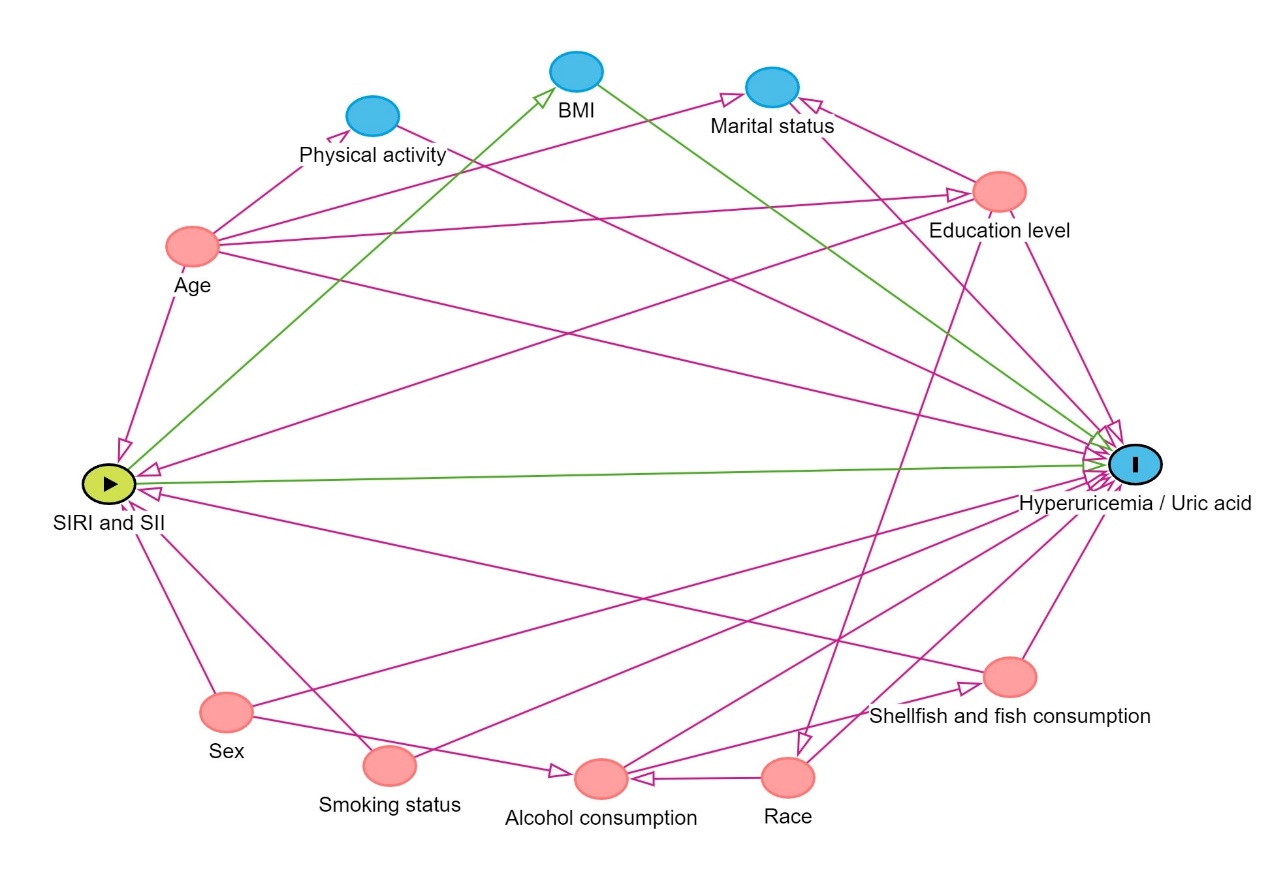


**Figure S1.** Directed acyclic graph (DAG) analysis for the associations of SII, SIRI with serum uric acid level and hyperuricemia risk.
